# Introducing high-cost health care to patients: dentists' accounts of offering dental implant treatment

**DOI:** 10.1111/cdoe.12129

**Published:** 2014-09-29

**Authors:** Christopher R Vernazza, Nikki Rousseau, Jimmy G Steele, Janice S Ellis, John Mark Thomason, Jane Eastham, Catherine Exley

**Affiliations:** 1 Centre for Oral Health Research, Newcastle UniversityNewcastle upon Tyne, UK; 2 Institute of Health and Society, Newcastle UniversityNewcastle upon Tyne, UK

**Keywords:** dental services research, prosthodontics, psychosocial aspects of oral health, qualitative research

## Abstract

**Objectives:**

The decision-making process within health care has been widely researched, with shared decision-making, where both patients and clinicians share technical and personal information, often being cited as the ideal model. To date, much of this research has focused on systems where patients receive their care and treatment free at the point of contact (either in government-funded schemes or in insurance-based schemes). Oral health care often involves patients making direct payments for their care and treatment, and less is known about how this payment affects the decision-making process. It is clear that patient characteristics influence decision-making, but previous evidence suggests that clinicians may assume characteristics rather than eliciting them directly. The aim was to explore the influences on how dentists' engaged in the decision-making process surrounding a high-cost item of health care, dental implant treatments (DITs).

**Methods:**

A qualitative study using semi-structured interviews was undertaken using a purposive sample of primary care dentists (*n* = 25). Thematic analysis was undertaken to reveal emerging key themes.

**Results:**

There were differences in how dentists discussed and offered implants. Dentists made decisions about whether to offer implants based on business factors, professional and legal obligations and whether they perceived the patient to be motivated to have treatment and their ability to pay. There was evidence that assessment of these characteristics was often based on assumptions derived from elements such as the appearance of the patient, the state of the patient's mouth and demographic details. The data suggest that there is a conflict between three elements of acting as a healthcare professional: minimizing provision of unneeded treatment, trying to fully involve patients in shared decisions and acting as a business person with the potential for financial gain.

**Conclusions:**

It might be expected that in the context of a high-cost healthcare intervention for which patients pay the bill themselves, that decision-making would be closer to an informed than a paternalistic model. Our research suggests that paternalistic decision-making is still practised and is influenced by assumptions about patient characteristics. Better tools and training may be required to support clinicians in this area of practice.

How clinicians and patients make decisions has been widely investigated but has been less well examined in dentistry 1,2. Well-defined patterns of decision-making have been described, although the boundaries between these apparently different patterns are not always clear. Charles et al. 3 in their key paper defined three types of decision-making, with the authors further refining this following empirical research 4. The authors describe a paternalistic style of decision-making, where the clinician possesses all of the technical knowledge with personal information from the patient either assumed or disregarded leaving the clinician to make decisions on the patient's behalf; an informed style where the clinician imparts all relevant technical information and the patient is the decision maker; a shared style where clinician and patient share information (both technical and personal) and then share the decision-making process. Although more shared approaches are generally favoured by Charles et al.*,* there is recognition that there should be flexibility in the style adopted, based on individual patient preferences.

These different styles recognize that patients hold information about personal preferences, social contexts, personal medical history and personal health beliefs, of which the clinician will not be aware, whilst the clinician holds information about the natural history of diseases, technical aspects of possible treatments and likely outcomes including side effects 5.

The paternalistic model which predominated until the 1970s 6 has now widely been discounted as a valid decision-making style as it fails to take any account of the patient held information and can therefore lead to inappropriate decisions 3,6,7. Additionally, there are also important political and professional imperatives directing a change away from a paternalistic model. In policy terms, using the UK as an example, there has been an increasing emphasis on patient choice with the concept embedded in National Health Service (NHS) policy 8,9 and a specific quality outcome to which NHS organizations are expected to adhere 10. There are also professional drivers; again using the UK as an example, the General Medical Council and General Dental Council both prescribe standards of informed consent in which patients should be involved in decision-making 11,12.

Despite all of this, the paternalistic model is still used, in at least some encounters, in dentistry 13 and health care more generally 14 as a result of barriers to introducing more shared styles. These may include insufficient time, a lack of tools or a lack of training to deliver information and to understand patient preferences 14 as well as the desire of clinicians to provide patients only with information about what they see as the ‘best’ treatment 13. There is also evidence that rather than patient preferences being ignored completely, clinicians often choose to assume patient preferences on the basis of other knowledge of the patient 15.

A wide range of personal patient-based attributes have been shown to influence clinicians in decision-making including financial status, socioeconomic status (SES), ethnicity, gender, age, education level and the personality characteristics of patients. Although some of these may influence the clinical outcome, often they will not 16. Such influences may not reflect patients' preferences or values at all, as illustrated in one study where clinicians offered patients of lower SES cheaper treatments, seemingly without eliciting patient preferences first 17. This has also been reflected in dentistry, with studies showing dentists were more likely to choose what may be viewed as simpler and inferior treatment options where patients had lower SES and more decayed teeth 18 or adjust their treatment plans based on their perceptions of patients' intelligence, attitude and/or emotional stability 19.

Although the (sometimes assumed) financial status of patients has been shown to influence doctors' decision-making, this is usually in a context where patients are not paying directly for their care (i.e. it is financed publicly or through insurance systems). In dentistry, direct user charges are common in health systems worldwide 20. Given this personal financial contribution to treatment, the actual cost of any treatment option is likely to shape patients' decision-making within dentistry 21 and one study has illustrated how patients may choose not to follow dentists' recommendations because of cost 13, in other words, cost clearly affects the patient in decision-making. However, it is not clear how cost influences the dentist in decision-making, for example, dentists may offer different treatments based on affordability to the patient or dentists may have a vested financial interest in selling more costly treatments. The use of direct charges shifts the clinician's role away from that of the gatekeeper who protects the resources of a third-party funder (such as a publicly funded healthcare system or an insurer), to a gatekeeper who would have a duty to only recommend health care which is actually needed by the patient (i.e. protecting the patient from themselves) which may also be in conflict with the dentists potential role as a business person with potential to benefit from the sale of such treatment. The potential conflict between acting in an ethical and professional way and the vested financial interest has been discussed and investigated thoroughly in the literature 22,23 most often in relation to the decision for dentists to practice in public or private systems 24,25. However, we do not know how patient charges, in particular for high-cost treatments, influences how the dentist participates in and undertakes decision-making.

The aim of this paper is therefore to explore the influences on how dentists engaged in a decision-making process where a large personal financial investment by the patient is usually involved. Specifically, we were interested in the initial decision whether and how to offer dental implant treatment (DIT) as an option for replacing missing teeth.

## Context

The treatment used as an exemplar in this study is dental implant treatment (DIT). DITs are a costly and relatively invasive dental management strategy used to substitute prosthetically for single or multiple missing teeth. In the context of this study (a UK setting), these are provided by dentists, generally with additional training and qualifications, working in primary care on a private basis. Nearly all patients receiving DIT generally pay a nonsubsidized (private) fee to the provider. For a very small group of patients with specific clinical conditions (such as maxillofacial reconstruction after oral cancer), DIT is available free of charge to the patient in NHS secondary care settings. Unless specifically stated, these results refer to the context of private provision of implants. Many of the dentists in this study did not provide implants personally but would refer patients on for DIT provision. Where this was the case, the interviewed dentist would receive no financial benefit from the patient accepting implants. Although DIT should be the minimum offered to edentulous patients as a first choice of treatment 26, missing teeth can also be replaced with dentures and bridges (both less costly and less invasive treatments) which can be provided under either private or NHS dental arrangements at a very much lower patient charge. The practising arrangements of each dentists were not specifically elicited, but inferences from interview data suggest that they worked under mixed (i.e. NHS and private) or private only conditions.

## Methods

These qualitative data were collected as part of a larger MRC funded study 27 which aimed to understand how clinicians and patients negotiate clinical need and treatment decisions within a context of finite resources, using the exemplar of dental implant treatment. The study had ethical approval from NHS Local Research Ethics Committee (Ref:06/Q0904/25) and was approved by the Research and Development arm of all the NHS trusts where a site was included.

### Sampling

All primary care dentists in one region of the North East of England were initially contacted with a postal questionnaire as part of a larger study published in full elsewhere 28. In brief, these quantitative findings suggested that provision of and referral for dental implants varied with age and sex of dentist. Included with the questionnaire was a ‘consent to contact’ form for dentists who were willing to be contacted about participation in a qualitative interview. Thirty-nine of the 209 questionnaire respondents completed a ‘consent to contact form’; these were more likely to be male, to offer DIT and to deliver DIT themselves than those who did not complete a form (see Table1). Purposive sampling was used to recruit responding dentists into the next, qualitative, phase of the study. The sample was selected to ensure a range of age, sex, socio-demographic practice settings^1^ and DIT providers and referrers.^2^. The inclusion of implant providers in our purposive sampling strategy meant that our sample is older, and more male than the underlying population of dentists. As data collection progressed, ‘snowball sampling’ was used to recruit additional dentists from groups whose views had not yet been sufficiently explored but where the available sample from the questionnaire respondents had been exhausted (chiefly young female dentists). Dentists were paid for the loss of time due to their involvement in the interviews at a nationally agreed rate.

**Table 1:** Characteristics of the sample

Category	Subcategory	Number in sample (*n* = 25) (%)	Among all questionnaire respondents (*n* = 204) (%)
Gender	Male	19 (76)	133 (65)
	Female	6 (24)	71 (35)
Age	<30	4 (16)	47 (23)
	30–39	5 (20)	51 (25)
	40–49	7 (28)	63 (31)
	50+	9 (36)	42 (21)
Implant provider status	Never	10 (40)	142 (70)
	Occasional	7 (28)	44 (21)
	Always	8 (32)	18 (9)

### Interviews

Written consent was gained from the participants. A focused interview was carried out by a single-trained interviewer (NR) at the participant's dental practice. Using a topic guide, the interviews covered the following broad topics: personal practice with regard to DITs, when and how DITs were discussed with patients, and whether and when referrals were made for DIT treatment. Earlier interviews informed the content of further interviews. Reflective field notes were made after the interview, to assist with analysis and to record any other information not gathered during the recorded interview. The interviews were digitally recorded, transcribed verbatim and transcripts anonymized. Demographic details about the participants were obtained from previous questionnaire results.

### Analysis

Thematic analysis based on the constant comparative method was carried out 29,30. Data collection and analysis occurred concurrently; emergent themes and issues from earlier interviews informed the content of subsequent ones. Data collection ceased when no new themes were being identified (‘saturation’). CE and NR (both social scientists) initially coded the data using a standardized agreed approach, and the wider research team (which included a health economist, dentists, a sociologist and a psychologist) participated in data sessions to discuss emergent codes. As data collection and analysis progressed, a coding frame was devised, tested and adjusted, and once refined applied to the transcripts using qualitative analysis software (NVivo7, 31). Subsequently, more detailed analysis was conducted by CV (a dentist by background), focussing specifically on dentists' accounts of how they introduced and discussed the option of DITs with patients.

## Results

Twenty-five interviews were carried out with 19 male and six female dentists. Quotes in the following section are labelled with the gender, age and provider status of each respondent. The provider status relates to their responses in the questionnaire, regarding whether they would always, occasionally or not ever personally provide dental implant treatment in the context of a scenario presented 28. Twelve interviewees provided at least some DIT themselves whereas 13 had no direct personal involvement in DIT. Interviewees ranged in age from 23 to 59. The characteristics of the sample are summarized in Table1.

The dentists' accounts suggest that there is significant variation in how they discussed or offered DITs with their patients. Dentists often made decisions about the appropriateness of implant treatment for a particular patient in advance of (and sometimes in the absence of) discussion with that patient. In some situations, patients raised the possibility of DITs themselves without prompting by the dentist. However, the way dentists reported dealing with such enquiries varied in a similar way to when dentist initiated (or failed to initiate) discussions. Analysis suggested that three approaches to discussing the treatment option of DITs could be described; labelled in this paper as ‘comprehensive’, ‘distorted’ and ‘incomplete’ presentation.

The first approach, ‘comprehensive’ presentation, was characterized by the dentist discussing implants as a potential treatment option, followed by the dentist explaining alternative options alongside the patient-specific indications and contraindications, sharing the decision-making process with the patient.

After [the consultation, I] write them this long letter which basically details why they came to see us, what their main concerns were, and what our examinations findings were and what the options of treatment were, and in most of the cases one of the options of treatment is doing nothing at all and leaving them as they are, and then it goes into the options of treatment and also how much is it going to cost. Male, 49, Always provider

The second approach, ‘distorted’ presentation, was characterized by dentists, having decided themselves that DITs were inappropriate, either following patient enquiries or independently, presenting information about treatment options in such a way as to reduce the chance of patients opting for DITs.

Before I got interested in implants I didn't really offer it to many people, I don't know why, I suggested it to them, said it was an option but that it cost a lot. And the way I was wording it to the patients it was as though I was putting them off, I realised that later on. Male, 26, Sometimes provider

The third approach also involved dentists deciding that DITs were inappropriate and then not discussing them as a treatment option with patients or, where patients made enquiries, the dentists dismissed them outright. This final approach, ‘incomplete’ presentation, also included a group who never considered implants as a treatment option (which may have either been due to them feeling that DITs were unsuitable for all of their patients or due to a lack of awareness about DITs as a potential treatment option).

I mean sometimes without doing it on purpose you sometimes do have preconceived ideas about people and whether they can afford something or whether it's something that they would be interested … I would have hoped to think I tried to say it but sometimes you do sort of accidentally eliminate something subconsciously I suppose. Female, 27, Sometimes provider

Although some dentists described using only one of these approaches, there was evidence that other dentists used different approaches in different situations. The analysis of interviews also did not identify any particular characteristic of the dentist that predisposed them to adopting one particular approach, but the questionnaire element of the larger study showed that older dentists were less likely to consider implants as a treatment option and that nonproviders working in practices where an implant provider was present were more likely to consider implants as an appropriate treatment option than nonproviders working in practices without implant providers present 28.

### Reasons for adopting particular approaches

The data suggest that some dentists were not able to describe, or were not overtly aware of, the approaches they adopted when discussing DITs with patients. However, other dentists explicitly discussed their approach and many appraised and reflected on their own approach within the interviews. This reflection is evident in the examples of ‘distorted’ and ‘incomplete’ presentation approaches already discussed. These reflections illuminated why certain dentists adopted particular approaches, and two major themes emerged from the analysis: direct drivers of behaviour and the influence of nonclinical attributes of patients. Each of these themes will now be explored in turn.

#### Motivations for behaviour: Business, professional and legal influences

Analysis revealed a number of influences on behaviour which could be broadly categorized as business, professional and legal. Very often, these three influences were in conflict with one another leaving the dentist with a difficult situation to resolve. A number of dentists noted their legal duties to explain and offer all treatment options in order to gain informed consent 11 and saw this as a driver for adopting the ‘comprehensive’ approach.

When you get into practice it [offering all options] is quite significant and one of the things that was really drummed into us was just how important it is to give your patients all of their options and put that in the notes so that legally they can't turn round later and say well you never gave me this as an option, and then they are in a position when they can, they can sue you or whatever, so yes I would always do it just for that and to cover your back as well. Female, 27, Occasional provider

Another dentist noted the professional obligation to offer all options, and reflects how this has changed over time, suggesting that he is more comfortable with a more paternalistic style.

It is in this new age thing that patients want dentists … to say, what do you recommend, tell me what to do. But in this day and age you know we can't do that, we just have to give people the options and all this woolly thing. I would much rather … give them the benefit of my knowledge and expertise … but we are not really supposed to say that nowadays. …. You tend to give them the benefits of each and sort of slant your argument a little bit in favour of the bridge. You tend to sort of give them the impression that you have arrived at a decision together whereas really you have prescribed the treatment for them. Male, 46, Occasional provider

However, dentists have another professional duty which is pertinent where the patient is paying for health care and especially when the health care is of high cost, and this is the need to avoid marketing or selling unnecessary treatments. Whilst trying to act professionally, there are therefore conflicts between offering full information on all available treatment options, and the need not to market and/or sell unnecessary treatments which will offer little or no clinical or health gain for the patient.

This conflict illustrates a different aspect of gatekeeping compared to that discussed in the introduction. Typically, the gatekeeper would be protecting the system funder (insurer or government) from unnecessary demand, whereas here the dentist may be acting as a gatekeeper preventing the patient themselves from incurring unnecessary expenditure (or simply health care). There is still a need for the typical gatekeeping role when considering the DITs provided by the NHS, although this will only apply to an extremely small number of cases, as noted by one dentist:

My view is they can't come in and demand, I would just say, look it's not justified. Umm. I mean that would be hard but the only thing is it's open to abuse. I suppose initially it is up to me but I would, I would only refer someone who I thought was appropriate. Female, 40, Never provider

Being a gatekeeper who protects patients may well be seen as part of the professional duty of a dentist when dealing with DITs. The analysis confirmed that this particular element was important to some dentists in adopting particular approaches, as shown in the example below.

I feel a degree of responsibility [for them spending their money], which is why if somebody says I want such and such doing, I'll say well, no I don't think that's a good idea. Male, 55, Never provider

For those dentists who are also acting as providers, the situation is even more complex as they may also stand to gain financially from providing implants, introducing another conflicting pressure. Interestingly, this situation was noted as a motivation for dentists to adopt ‘incomplete’ presentation of DITs as a form of overcompensation so that they could not be perceived as overselling DITs and therefore profiting from a high-cost intervention. One provider noted this conflict (albeit by drawing an extreme dichotomy), but framed the problem in terms of what the patient would think rather than the internal conflict for the dentist.

The problem being a health professional and balancing it with the business aspect of it, nobody goes into business other than to make profit. Nobody goes into a health profession other than to help people, so striking the balance is quite a difficult thing and one has to do it conscious of the patient's autonomy and their self-determination and ability to decide … if you infringe upon that a lot of patients, even if they were happy to go ahead, might say ‘I am sorry I think he is pushing me into it.’ Male, 49, Always provider

The analysis therefore confirms that business, professional and legal duties have an important role in how high-cost treatments are offered, but this is a complex situation with different conflicts occurring.

#### Perceived attributes of patients

It has long been recognized the personal characteristics of patients should form an important element of decision-making 4. The characteristics can be divided into those that have a direct impact clinically (i.e. those that would affect the outcome of treatment) and those that are nonclinical (i.e. those that affect the decision-making process but would not have an impact on the outcome of treatment).

Although clinical contraindications (for example, a lack of sufficient bone to place DITs or medical problems complicating surgery) could have been a legitimate reason to exclude DITs from the decision-making process, many of the dentists interviewed recognized that there were very few absolute contraindications to DITs, therefore discounting this as a major driver behind ‘incomplete’ or ‘distorted’ presentation approaches.

I think it is an option for pretty much anybody … I mean obviously there are more risks attached to it in people with heavily resorbed ridges, but it is still an option you know that should be offered. Male, 26, Occasional provider

DITs were, however, excluded by those adopting the ‘distorted’ or ‘incomplete’ approaches, and in these cases, nonclinical patient attributes were a frequently discussed as potential drivers for this exclusion. Specific attributes occurring most frequently in the analysis were perceived motivation for extensive dental treatment and whether patients valued their teeth as well as financial impacts on decisions (or ability to pay).

As noted above, these nonclinical attributes are all important elements of patients' preferences and values and should form an important part of shared decision-making. However, where dentists did mention these attributes as a factor in their decision-making process, it was as a reason for adopting the ‘distorted’ or ‘incomplete’ approaches. In particular, using such approaches was justified in terms of avoiding unnecessary wasting of time and resources of providers and (where affordability was concerned) to avoid embarrassing the patient by offering what was assumed to be an unaffordable treatment.

I'm only going to refer the people who I think are actually going to go ahead with all this, and the people you think well, you're quite likely to duck out half way through the treatment and waste everybody's time, so they've got to be pretty keen really. Male, 36, Never provider

I would probably mention it but I would do it in a way that they didn't feel embarrassed. I would probably tell them that I do realize it's very expensive and this isn't something that is for everyone because most people can't afford it, so I wouldn't make them feel that, oh they had to somehow skimp and save to actually find this sort of money, because I mean it is a lot of money. Male, 57, Never provider

Using inferred nonclinical attributes in this way can limit the patient's role in the decision-making process which may, in turn, mean that dentists are not fulfilling their professional obligations.

### Assessing nonclinical attributes

Further analysis of the interviews with dentists using nonclinical attributes in decision-making led to interesting findings relating to how the attributes were elicited for each patient. In many instances, dentists talked either implicitly or explicitly about making assessments about these attributes based not on discussion with patients but based on their previous knowledge about the patient, knowledge of some demographic details or physical (including dental) appearances.

Assumptions were made about motivation for the invasive and lengthy treatment involved in DITs based around lack of patient complaints about their oral health, other health problems and age. In some instances, evidence of previous treatment provided was also used as an indicator of future motivation. Many dentists presumed that if a patient did not mention any problems that there was no need to offer implants, as the patient was not motivated to have this treatment. This attitude may have been adopted by the dentist in order to mitigate the conflicts explored earlier relating to marketing unnecessary treatments.

If a patient comes and they, you know, you say how are you managing with your teeth, oh, they're fine, you know, why should I suggest an implant? Female, 56, Never provider

Further examples suggest that dentists used attendance patterns and previous treatment decisions as well as how patients looked after their mouths to help them decide whether or not the patient valued their teeth and/or oral health sufficiently to be interested in DITs, rather than a direct discussion with patients, as illustrated in the next quote:

The [patient] attitude is a big factor for me, you know, if somebody has got a really bad attitude to their oral health I am not bothered about them. You know what is the point of trying to help them, they don't want to help themselves, they don't even turn up for their appointments half the time. Male, 26, Occasional provider

In addition to motivation, known or visible attributes such as where the patient lived and their appearance appeared to affect dentists' perceptions about whether a patient was likely to be able to afford implants or not. The first quote below illustrates where one dentist assumed ability to pay incorrectly albeit for bridges rather than implants (and also illustrates a form of the ‘distorted’ approach to offering treatments by over-quoting prices). The second and third quotes illustrate the use of appearance and where the patient lived to assume ability to pay.

When I first took on the practice I had a lady come in who wanted … multiple bridge work and she was on income support. I said this couldn't be done under the health service and I actually quoted a ‘get lost’ figure … A lot of money, to put them off. She came back five weeks later with the cash and asked if I would start. I subsequently learned that she was a ‘professional lady’ and she must have worked very hard for a few weeks. Male, 55, Never provider

To look at these people you would not think they were able to have that sort of money … One of my first ever implant cases, was a lady who used to come in a tatty old coat, buttons missing and fraying edges everywhere and I gave her the option, I must admit I probably didn't give her the option properly, but she [said] ‘oh, that makes sense and how much, yes fine.’ Male, 41, Always provider

I would tell them about implants but I would have to tell them that, well I do realize that you have sort of just come from the council estate [social housing] across the road … I wouldn't actually say I realize you have come from the council estate, but I would know the address and therefore I would know what sort of house they came from and therefore what sort of disposable income they probably had. Male, 57, Never provider

In all of these instances, the patients' preferences were therefore inferred or assumed rather than elicited explicitly. Occasionally, the assumptions were taken further and there was evidence that patients were sometimes labelled in a judgemental way, such as in the following example where patients are labelled as good or bad based on their treatment preferences.

Good patients are the patients who go for root treatments and not extractions, and are here for their 3 month recalls and are keen on a dentist, not keen on my dentistry but keen to keep teeth, will do what's necessary to keep teeth rather than what we see a lot of unfortunately cos there's a lot of people out there who just come in and say just take it out, not bother, turn up every three years, come in in pain and just want it fixed, that would probably count as a good patient or a bad patient. Male, 36, Never provider

Of those dentists that made assumptions, some openly said that they were aware of doing this and recognized that it was not ideal. However, others, whilst demonstrating that they did make assumptions, did not see this as a problem in the interview, or were perhaps not aware that they were making assumptions.

## Discussion

This study set out to explore the different influences on how dentists decided whether or not to introduce a treatment option where a large personal financial investment by the patient is usually involved, in this instance DITs, and, if they did, how they engaged in the decision-making process. From the analysis of interviews with dentists, three approaches to offering implants were noted: ‘comprehensive’, ‘distorted’ and ‘incomplete’ presentation. Further exploration of the influences on which approaches are adopted suggested that awareness of decision-making styles, business, professional and legal obligations and patient characteristics were important. Interestingly, although patient characteristics were used by many dentists, these were usually not elicited directly from the patient but assumed based on patient appearances or demographic details.

Although the findings have been discussed and explored within the results section, it is worth considering the applicability and influence of the methodology on the results and the context of these findings in the field alongside their implications here.

The analysis presented here is based on dentists' accounts and there may have been a temptation to discuss only those perspectives felt to be acceptable in the context of a discussion about professional practice. However, the data and the interviewer's (NR) impression suggest that the interviewees were notably candid about their practice and their attitudes to their patients.

The analysis was conducted principally by three of the authors, two of whom are medical sociologists with limited dental knowledge and one further academic paediatric dentist whose practice does not include any DIT related aspects. On this occasion, this ‘outsider’ status appears not to have restricted access to dentists accounts and may have enabled the researchers to ask questions about aspects of care that would be ‘taken for granted’ by those within primary care dentistry. Additionally, reflexivity (i.e. the researchers own perspective and influences on the analysis) was explored and challenged in data workshops and discussions with other members of the team.

The context of the study was one region, the North East, of England. Primary care dentistry in England has undergone significant changes in the last fifteen years, with a very substantial expansion of private treatment. Dentists (and patients) in this study were still adjusting to an expanding demand and market for DITs, especially in the region concerned; few will have been prepared for this in their dental training. Additionally, the sample was purposively selected and this led to an over-representation of older, male dentists. Our analysis did not suggest that this group were particularly linked to the practices discussed, but this should be borne in mind when interpreting these findings. In general, our findings should have relevance to other mixed economy dental healthcare settings.

This study provides an important insight into different approaches to offering treatments in dentistry. Although research on decision-making in dentistry is a growing field, recent research has tended to focus on the use of decision aids 32 and the patient perspective 33 or clinical influences 34; there is paucity of research on the dentist's approach to decision-making. The persistence of a paternalistic style is a common finding from research in other areas of health care 14, and this finding is replicated in this study. What marks this area of dentistry out as different is that patients are paying directly for treatments. While it might be expected that in this context there would be greater patient involvement in decision-making there is some evidence from the data that, conversely, this may be a reason for retaining paternalism. For a dentist who is acting in a gatekeeper role (rather than being a provider) using a paternalistic style may make it easier for them to fulfil their professional duty to protect the patient from high personal costs. In the case of providers, there is evidence that some use paternalism to protect themselves from the business influences on decision-making and to uphold professional responsibilities not to over-sell treatment.

Even when patients initiate discussions around DITs, which might suggest an informed or patient-led consultation, there is evidence that some dentists still present information in a ‘distorted’ or ‘incomplete’ way pushing the process back towards paternalism. Indeed, this suggests that although the categories of presentation defined in this paper fit with the categories of decision-making defined by Charles et al. 4 they do not describe the same things and the categories of presentation actually work across the Charles et al. categories.

These findings sit within a literature relating to the conflicts between professional duty and business practice which although present in general medical practice 35 is likely to be more acute in general dental practice 23. In general, it has been found the professional duty usually has a stronger influence than business or economic aspects 36,37 and the findings of this study echo this with dentists using paternalistic styles of decision-making to protect themselves from business influences and uphold professional duties. However, this raises an important ethical question, as operating a shared decision-making style is also a professional duty and so in protecting one professional duty (not over-selling treatment), another is being disregarded. Ideally then, upholding the professional duty not to over-sell should not be performed by adopting a paternalistic style of decision-making.

A persistence of a paternalistic style is at odds with the desire for increased patient autonomy and may mean that any consent is not fully informed. There is an opportunity to move away from paternalistic styles if appropriate tools are available. For dentists in a provider role, the use of proven tools for shared decision-making may actually be highly desirable, providing a way of avoiding the ‘selling’ of interventions. Better tools for eliciting and discussing patient preferences, developed using tested scientific approaches may therefore be helpful in dentistry, such as those already available in other areas of medicine 38. The development of such tools for DITs and further work to investigate the influence of these on decision-making are important areas for future research.

An additional area of concern is the assumptions made by dentists about patients, which in turn influence decision-making. The assumptions revealed in the interviews were very similar to those shown by others investigating influences on dental decision-making for other types of treatment 19,39, and nonclinically relevant characteristics have been found to influence treatment provided in other areas of dentistry 40,41 as well as health care more generally 42. The reasons for such assumptions were not obvious from the analysis undertaken here and there is only partial recognition of this as a potential problem by dentists. This could present a major ethical challenge as this behaviour could disadvantage patients by limiting what is offered to them on the basis of assumed characteristics. To begin to address this problem, a greater understanding of the reasons for such assumptions, as well as a quantification of the size of the problem is necessary and this is an area for future research.

## Conclusion

These data suggest that when making decisions about a high-cost dental intervention (DITs) where the patient meets the costs directly, shared decision-making is limited. Instead, some dentists do not offer DITs or distort the information provided to influence the decision-making process. When influences on the approach adopted are analysed, some dentists are explicitly aware of adopting particular approaches but professional obligations and patient characteristics are also important. Interestingly, the characteristics of motivation for treatment and ability to pay are often assumed based on appearance or demographic details. This is an important finding with ethical implications worthy of further research.

Dentists should be aware of the decision-making styles they are adopting and assumptions they make, and it is important to encourage dentists to reflect on their own practice. However, even with insight, there are a number of difficult conflicts. Decision-making tools specific to dentistry would be one way of minimizing these conflicts to aid in this area and further research on their use is necessary.

## Notes

* The questionnaire did not collect formal data on the socio-demographic characteristics of practices; however, we used our informal knowledge of the study area to select practices in affluent and deprived areas; and in a range of geographical locations including market towns, city centre, suburban etc.

† DIT providers are older and more likely to be male than dentists who are not DIT providers 28
